# Fatty Acid Profiles in Tissues of Japanese Eel (*Anguilla japonica*) from the Yangtze Estuary and Adjacent Offshore Waters: A Comparative Analysis

**DOI:** 10.3390/ani16162568

**Published:** 2026-08-18

**Authors:** Chao Song, Dongyu Song, Chengyao Yang, Yijia Li, Hang Li, Zhiqiang Ye, Junlin Ren, Sikai Wang, Feng Zhao

**Affiliations:** 1 National Engineering Research Center for Marine Aquaculture, Zhejiang Ocean University, Zhoushan 316022, China; songc@ecsf.ac.cn (C.S.); 19818007280@163.com (D.S.); 2 East China Sea Fisheries Research Institute, Chinese Academy of Fishery Sciences, Shanghai 200090, China; ychydx666@163.com (C.Y.); liyijia0412@163.com (Y.L.); 13376366880@163.com (H.L.); j8954871@163.com (Z.Y.); renjl@ecsf.ac.cn (J.R.); wangsk@ecsf.ac.cn (S.W.); 3 School of Environment and Architecture, University of Shanghai for Science and Technology, Shanghai 200093, China; 4 College of Fisheries and Life Science, Dalian Ocean University, Dalian 116023, China; 5 College of Marine Sciences, Fujian Agriculture and Forestry University, Fuzhou 350002, China

**Keywords:** Yangtze Estuary, offshore, *Anguilla japonica*, fatty acid, distribution patterns, nutritional requirements

## Abstract

To compare the fatty acid composition in different tissues of *Anguilla japonica* before and after sea entry, we analyzed the muscle, liver, and ovary of eels from the Yangtze Estuary and offshore waters, and describe the distribution patterns of various fatty acids in different tissues. Comparing different tissues, in eels from the estuary, the muscle had the highest content of EPA, the liver had the highest content of DHA, and the ovary had the highest contents of ARA. In offshore eels, the muscle had the highest contents of C16:1 and C18:1n9c; the liver had the highest content of C16:0; and the ovary had the highest contents of C18:0 and DPA3. Comparing eels before and after sea entry, the contents of C14:0, C16:0, C20:1n9 and C20:2 in the ovary of estuary eels were significantly higher than those in the offshore eels. Conversely, the contents of C18:0, C20:0, DPA3, HUFA, total n3-PUFA and n3/n6 ratio in the ovary of offshore eels were significantly higher than those in estuary eels. Ovaries of estuarine eels showed higher proportions of saturated fatty acids, whereas ovaries of offshore eels showed higher proportions of n3-PUFA. The observed differences in ovarian fatty acid profiles may reflect the nutritional demands associated with gonadal development stages, which were determined by histological oocyte staging.

## Simple Summary

*Anguilla japonica* is a catadromous species that migrates to the ocean for spawning. By comparing the fatty acid composition in different tissues of eels collected from the Yangtze Estuary and adjacent offshore waters—presumed to represent distinct migratory stages based on capture location and gonadal maturity—we observed distinct distribution patterns. In estuarine eels, muscle and liver tissues were dominated by fatty acids associated with energy supply and hepatic metabolism. In contrast, ovaries of offshore eels exhibited significantly higher proportions of n3-HUFA. These site- and tissue-specific differences suggest a potential preferential allocation of essential fatty acids to support ovarian development during seaward migration. Our findings provide baseline descriptive data that may contribute to future research on the reproductive physiology and conservation of this species.

## 1. Introduction

*Anguilla japonica* is a catadromous species widely distributed across major Asia river systems, including coastal waters, estuaries, and inland lakes in China [1]. Each spring, glass eels migrate from the sea into estuaries and ascend rivers, where they reside, grow, and accumulate energy reserves in various freshwater habitats. Feeding intensity peaks during summer and early autumn, when eels prey mainly on invertebrates (e.g., crabs and oligochaetes) and small fishes. As they mature and undergo silvering in late autumn, they cease feeding, with digestive organs gradually degenerating, and begin their seaward spawning migration [2,3,4]. Under natural conditions, gonadal development relies heavily on the physiological and nutritional status acquired from prey organisms prior to migration [5]. Since broodstock nutrition—particularly the composition of essential fatty acids—critically influences reproductive performance and larval quality [6,7], understanding the mobilization of stored nutrients before sea entry is fundamental to evaluating subsequent reproductive success. However, the specific dynamics of lipid redistribution across tissues during this non-feeding migratory phase remain poorly understood.

While substantial research on *A. japonica* has focused on nutritional requirements in aquaculture [8,9], rearing techniques [10], artificial reproduction [11], and larval cultivation [12,13], field-based studies have gained increasing attention in recent years due to population declines caused by dam construction, habitat degradation, and overfishing. Consequently, surveys of wild populations [14], migratory behavior [15], and spawning ecology [16,17] have been conducted to support conservation efforts. Regarding the nutritional status of wild eels, previous studies have examined adult eels from rivers, bays, and spawning grounds [18,19], compared captive-reared versus wild silver eels during artificial maturation [20,21], and analyzed fatty acid profiles in glass eels [22]. However, these studies have primarily focused on single tissues or particular life stages. To date, no investigation has systematically compared the tissue-specific distribution (simultaneously in the muscle, liver, and ovary) of fatty acids between immediate pre-migratory (estuarine) and early post-migratory (offshore) wild silver eels, a critical gap given that stored lipids fuel the non-feeding spawning migration.

The Yangtze Estuary is a critical passageway for the seaward spawning migration of *A. japonica*. Previous studies have shown that eels in the estuary generally have ovaries at stage II before entering the sea, with gonads continuing to mature after migration [3]. However, little is known about their migratory routes, distribution areas, residence time in offshore waters, or whether they continue feeding to replenish energy reserves during this non-feeding migratory phase. Moreover, the energy reserves and nutritional status of eels with stage-II gonads in the Yangtze Estuary remain unclear, and the changes in tissue-specific fatty acid distribution following seaward migration have yet to be characterized. Given that fatty acid profiles directly reflect tissue functions and the respective roles of fatty acids in energy supply and nutritional storage [23,24], this study compared fatty acid compositions in the muscle, liver, and ovary of *A. japonica* collected from the Yangtze Estuary and adjacent offshore waters. The specific objectives were (1) to characterize tissue-specific fatty acid distribution patterns in the two groups and (2) to determine whether the observed differences indicate preferential allocation of essential fatty acids to support ovarian development during seaward migration.

To minimize confounding effects from non-migratory individuals and seasonal variation, all samples were collected during the same peak migratory period, ensuring that the specimens had comparable age distributions [25]. Histological examination confirmed that estuarine eels had ovaries at single-layered follicle stage (stage II), whereas offshore individuals had progressed to adipose vesicle stage (stage III) [26] (Figure S1). Otolith Sr/Ca profiles verified their catadromous life history and further revealed their migratory stage at capture: estuarine eels were migrating from freshwater to the estuary, while offshore eels had migrated from freshwater through the estuary to offshore waters (Figure S2). Notably, offshore individuals had empty stomachs, consistent with the cessation of feeding during migration. This integrative sampling and verification strategy allowed us to compare fatty acid composition differences between pre- and post-entry eels in the context of gonadal development. The study provides baseline tissue-specific fatty acid profiles, which may inform future assessments of reproductive potential and nutritional dynamics during seaward migration. We acknowledge that individual variation and environmental conditions may still influence the observed profiles; therefore, our findings should be interpreted as descriptive and hypothesis-generating, warranting further validation through controlled experiments.

## 2. Materials and Methods

### 2.1. Animals and Sampling

**Figure 1:**
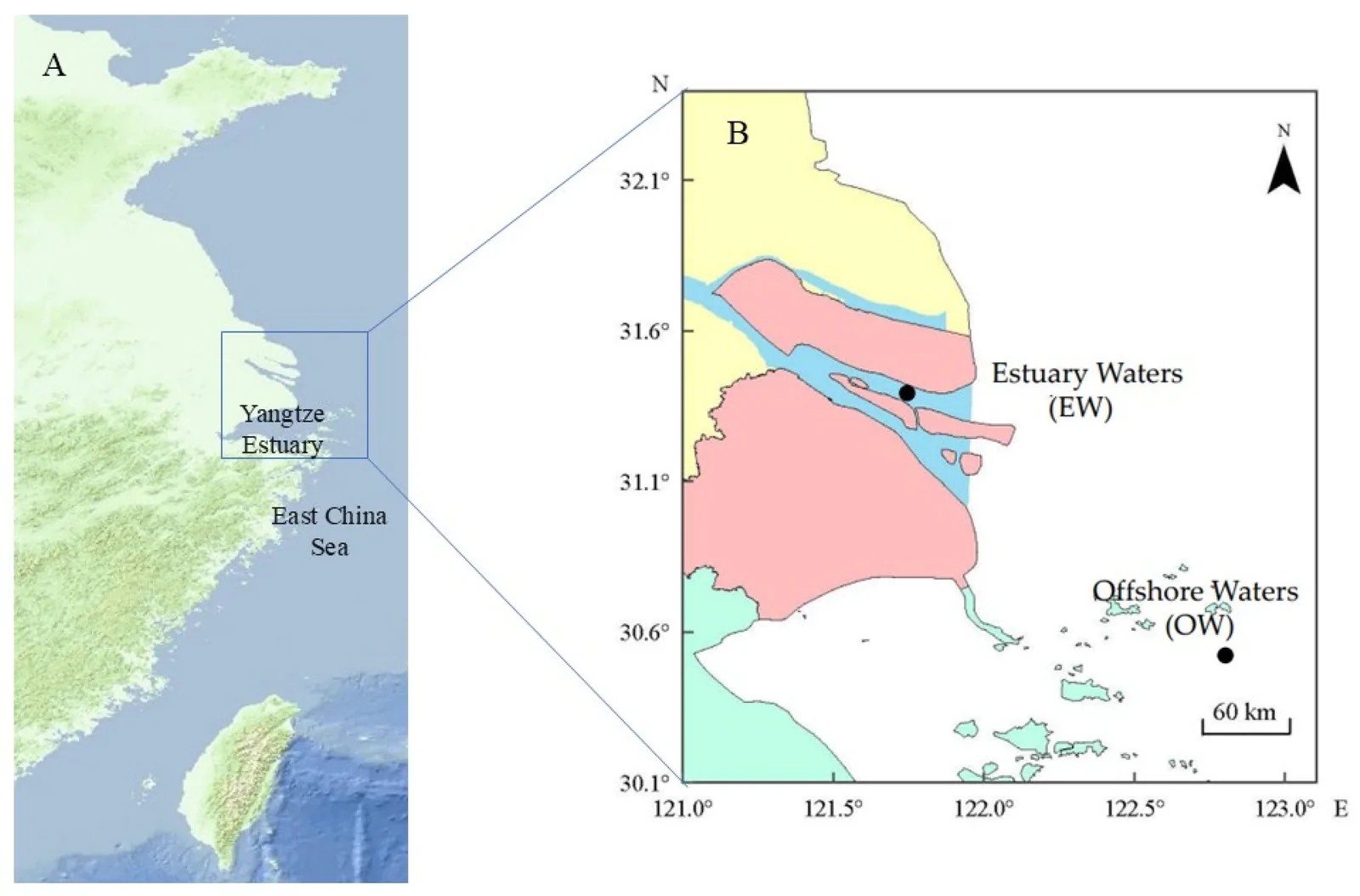
Japanese eel (*A. japonica*) sampling waters. (**A**): Sampling area. The box indicates the sampling area distributed in the Yangtze River estuary and the coastal waters of the East China Sea. (**B**): Sampling waters, including the Yangtze River estuarine waters (EW) and offshore waters (OW).

Samples were collected in November, during the peak seaward migration season, from both the Yangtze Estuary (EW, approximately 31°26′ N, 121°47′ E) and adjacent offshore waters (OW, approximately 30°33′ N, 122°40′ E), to obtain individuals representing the pre-entry (estuarine) and post-entry (offshore) migratory stages, respectively (Figure 1). All specimens were caught by local fishermen using hook and line and were purchased on site. Upon collection, the sex of each individual was determined by macroscopic observation of gonadal morphology and further confirmed by histological examination of gonadal tissue sections [26]. Six female individuals were randomly selected from each sampling site for fatty acid composition analysis. The mean body length and weight of the EW eels were 60.90 ± 4.78 cm and 401.78 ± 158.81 g, respectively, whereas those of the OW eels were 72.38 ± 4.86 cm and 681.49 ± 79.03 g, respectively.

Before fatty acid analysis, we assessed the gonadal developmental stage and migratory background of the selected specimens. Gonadal stages were classified according to ovarian morphology and oocyte development [26]. Stage II ovaries, characterized by oocytes with a single-layered follicle epithelium, were defined as the single-layered follicle (SLF) stage; stage III ovaries, marked by the appearance of adipose vesicles, were defined as the adipose vesicle (AV) stage (Figure S1). Accordingly, estuarine eels had ovaries early at Stage II, with low gonadosomatic index (GSI) values (0.42 ± 0.38). In contrast, offshore eels had progressed to Stage III, with significantly higher GSI values (1.61 ± 0.47). To further confirm the migratory origin of the samples, otolith microchemical analysis was performed on a subset of individuals [15]. The results indicate that both EW and OW eels are migratory individuals, captured in different habitats: EW eels were sampled in the estuary after freshwater emigration, while OW eels were sampled in offshore waters after passing through the estuary (Figure S2).

### 2.2. Sample Processing and Analysis

Samples were collected from the Yangtze Estuary (estuarine group, EW) and adjacent offshore waters (offshore group, OW). Muscle, liver, and ovary tissues were dissected on an ice-cooled plate, placed in sealed plastic bags, transported on dry ice to the laboratory, and stored at −20 °C until analysis. Total lipids were extracted from approximately 2 g of homogenized tissue using chloroform–methanol (2:1, *v*/*v*, 30 mL) containing 0.01% (*w*/*v*) pyrogallic acid as an antioxidant, according to the Folch method. After homogenization, the mixture was washed with 6 mL of 0.9% (*w*/*v*) NaCl solution, and the lower organic phase was collected. The upper aqueous phase was re-extracted with 10 mL of chloroform. The combined organic phases were dried over anhydrous Na_2_SO_4_ and evaporated to dryness under a gentle stream of nitrogen at 40 °C until constant weight. The extracted lipids were stored at −20 °C.

Approximately 50 mg of total lipid was transferred to a screw-capped tube and saponified with 2 mL of freshly prepared 0.5 M sodium hydroxide in anhydrous methanol at 80 °C for 10 min with intermittent vortexing until complete dissolution. After cooling, 2 mL of 15% (*w*/*w*) boron trifluoride (BF_3_)–methanol complex was added, and the mixture was heated at 80 °C for 10 min to complete methylation. The reaction was terminated by cooling on ice; then 2 mL of n-heptane and 2 mL of saturated NaCl solution were added sequentially. The upper heptane layer containing fatty acid methyl esters (FAMEs) was collected, dried over anhydrous Na_2_SO_4_, and concentrated under a gentle nitrogen stream to a final volume of 1 mL for gas chromatography (GC) analysis. FAME analysis was performed on an Agilent 6890 gas chromatograph (Agilent Technologies, Santa Clara, CA, USA) equipped with a flame ionization detector (FID) and a SP-2560 fused-silica capillary column (100 m × 0.25 mm i.d., 0.20 μm film thickness; Supelco, Bellefonte, PA, USA). The injector temperature was set at 250 °C with a split ratio of 20:1; the injection volume was 1 μL. The detector temperature was 260 °C. Nitrogen was used as the carrier gas at a constant flow rate of 1.0 mL/min. The oven temperature program was as follows: 140 °C held for 5 min, then increased to 240 °C at 4 °C/min and held for 20 min (total run time 50 min). FAMEs were identified by comparison of retention times with a certified Supelco 37-component FAME standard mixture (CRM47885). A reference standard mixture was injected every 20 samples to monitor retention time and peak area stability. The relative proportion of each fatty acid was calculated by area normalization and expressed as a percentage (%) of the total area of all identified fatty acid peaks, after excluding solvent and unresolved peaks. No response correction factors were applied for FID.

### 2.3. Statistical Analysis

Statistical analyses were performed using SPSS 25.0 (IBM Corp., Armonk, NY, USA). Normality was assessed using the Shapiro–Wilk test, and homogeneity of variances was examined using Levene’s test for each tissue type when comparing estuarine (EW) and offshore (OW) groups. Based on these results, independent-sample *t*-tests (with Welch’s correction applied when variances were unequal) or Mann–Whitney U tests were used as appropriate. For comparisons among three tissues (muscle, liver, and ovary) within each source group, repeated-measures ANOVA was performed, with individual fish treated as the repeated factor. Residual normality and sphericity were assessed; when the sphericity assumption was violated (Mauchly’s test, *p* < 0.05), the Greenhouse–Geisser correction was applied to adjust the degrees of freedom. When a significant main effect was detected in the repeated-measures ANOVA, post hoc pairwise comparisons were conducted using paired-sample *t*-tests with Bonferroni correction for multiple testing. Bonferroni correction was chosen to strictly control the family-wise error rate (FWER), given the limited number of pairwise comparisons (*n* = 3). All data are expressed as mean ± standard deviation (SD), and the significance threshold was set at *p* < 0.05 for all tests.

## 3. Results

### 3.1. Difference of Fatty Acid Composition Before and After Seaward Migration

A total of 32 fatty acids (12 SFA, 9 MUFA, 11 PUFA) were detected in the muscle (Table 1). The predominant fatty acids in both groups were C16:0, C18:1n9c, C18:2n6c, and DHA. Significant differences between EW and OW were observed for the fatty acids and derived indices, including C12:0, C15:1, C18:1n9t, C22:1n9, C20:2, the PUFA content and PUFA/SFA ratio (*p* < 0.05). The contents of EPA+DHA, HUFA, and n3-PUFA in muscle were higher in EW than in OW, whereas MUFA content showed the opposite trend (Table 1).

A total of 30 fatty acids (12 SFA, 7 MUFA, 11 PUFA) were detected in the liver (Table 2). The predominant fatty acids in both groups were C16:0, C18:1n9c, and DHA. Significant differences between EW and OW were observed for the fatty acids and derived indices, including C16:0, C18:1n9t, C20:1n9, C22:1n9, the SFA content, and DHA/EPA ratio (*p*< 0.05). The contents of EPA + DHA, HUFA, and n3-PUFA in the liver were higher in EW than in OW, whereas SFA content showed the opposite trend. The DHA/EPA, EPA/ARA, n3/n6, and PUFA/SFA ratios in the liver were all higher in EW than in OW (Table 2).

A total of 24 fatty acids (8 SFA, 7 MUFA, 9 PUFA) were detected in the ovary (Table 3). The predominant SFA in EW was C16:0, whereas in OW it was C18:0; C18:1n9c was the most abundant MUFA in both groups. Significant differences between EW and OW were observed for the fatty acids and derived indices, including C14:0, C16:0, C18:0, C20:0, C20:1n9, C20:2, DPA3, the HUFA, n3-PUFA contents, and n3/n6 ratio (*p* < 0.05). The contents of HUFA and n3-PUFA in the ovary were lower in EW than in OW, whereas SFA content showed the opposite trend. The n3/n6 and PUFA/SFA ratios in the ovary were lower in EW than in OW, while the DHA/EPA ratio was higher (Table 3).

### 3.2. Difference of Fatty Acid Composition Among Different Tissues

**Figure 2:**
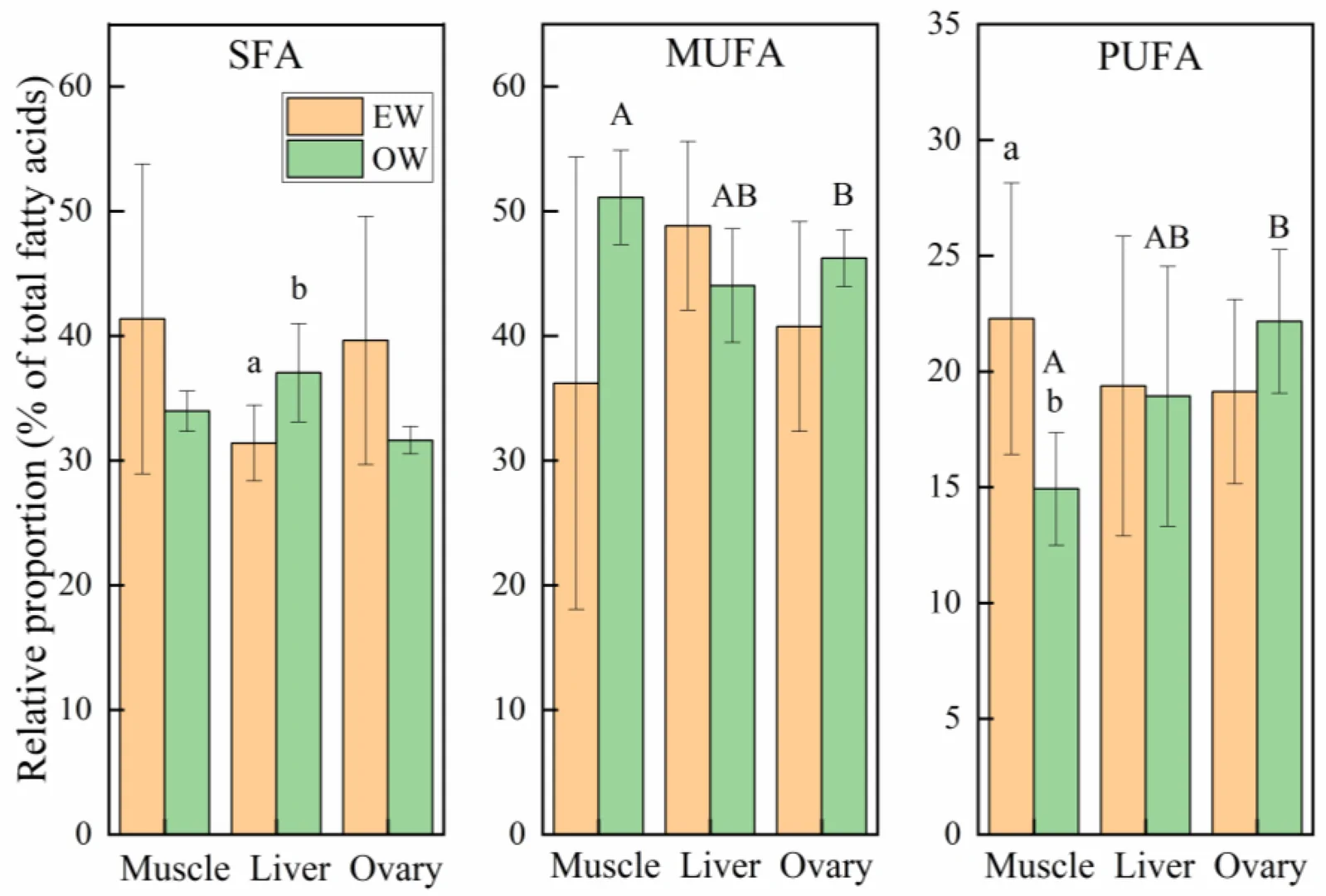
Comparison of the total content of different types of fatty acids (SFA, MUFA, PUFA) in different tissues of Japanese eels (*A. japonica*) from EW and OW. EW: estuary waters; OW: offshore waters. Error bars represent standard deviation (SD). Different small letters (a, b) denote significant differences between samples from different waters of EW and OW. Different capital letters (A, B) denote significant differences among different tissues of muscle, liver and ovary.

Figure 2 shows the fatty acid composition across different tissues of *A. japonica* from EW and OW. For SFA, the highest content was in the muscle and lowest in the liver for EW individuals, with no significant inter-tissue differences (*p* > 0.05); in contrast, for OW individuals, the highest was in the liver and lowest in the ovary. For MUFA, EW fish had the highest content in the liver and lowest in the muscle with no significant inter-tissue differences (*p* > 0.05), whereas OW fish showed the highest in muscle and lowest in the liver, with muscle content significantly higher than that in the ovary (*p* < 0.05). For PUFA, EW fish exhibited the highest in the muscle and lowest in the ovary, with no significant inter-tissue differences (*p* > 0.05), while OW fish had the highest in the ovary and lowest in the muscle, with muscle content significantly lower than that in ovary (*p* < 0.05).

**Figure 3:**
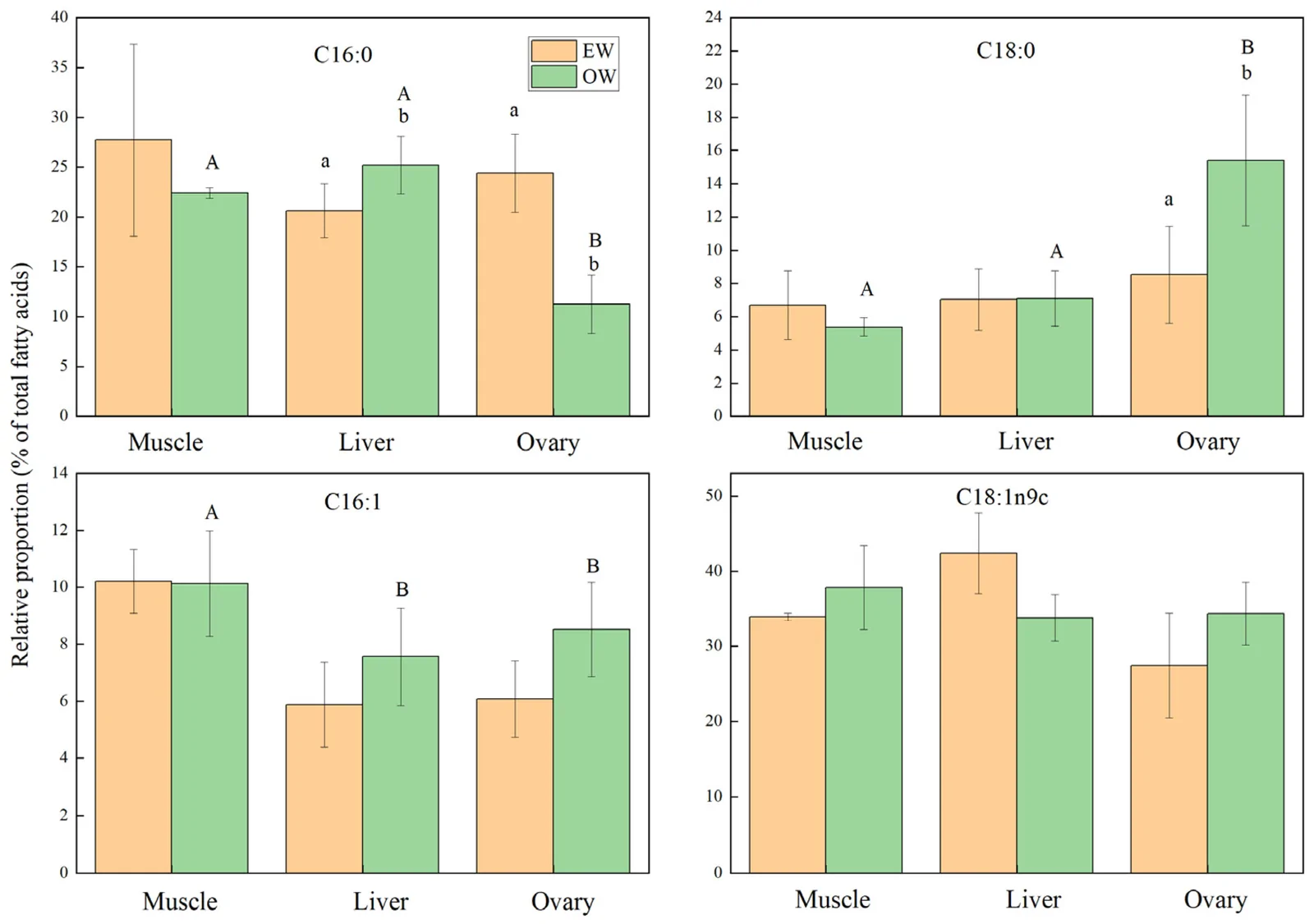
Comparison of the main fatty acid contents of SFA (C16:0 and C18:0) and MUFA (C16:1 and C18:1n9c) in different tissues of Japanese eels (*A. japonica*) from EW and OW. EW: estuary waters; OW: offshore waters. Error bars represent standard deviation (SD). Different small letters (a, b) denote significant differences between samples from different waters of EW and OW. Different capital letters (A, B) denote significant differences among different tissues of muscle, liver and ovary.

Figure 3 presents the contents of the main SFA (C16:0 and C18:0) and MUFA (C16:1 and C18:1n9c) in different tissues of *A. japonica* from EW and OW. For C16:0, EW fish showed the highest content in the muscle and lowest in the liver, with no significant inter-tissue differences (*p* > 0.05); in contrast, OW fish had the highest content in the liver and lowest in the ovary, with ovary values significantly lower than those in liver and muscle (*p* < 0.05). For C18:0, both groups exhibited the highest content in the ovary and the lowest in the muscle; moreover, the ovarian C18:0 content was significantly higher in OW fish than in EW fish (*p* < 0.05). For C16:1, both groups showed the highest content in the muscle and the lowest in the liver; additionally, in OW fish, the muscle C16:1 content was significantly higher than that in the liver and ovary (*p* < 0.05). For C18:1n9c, EW fish had the highest content in the liver and lowest in the ovary, whereas OW fish had the highest in the muscle and lowest in the liver; no significant differences were found among tissues within either group (*p* > 0.05).

**Figure 4:**
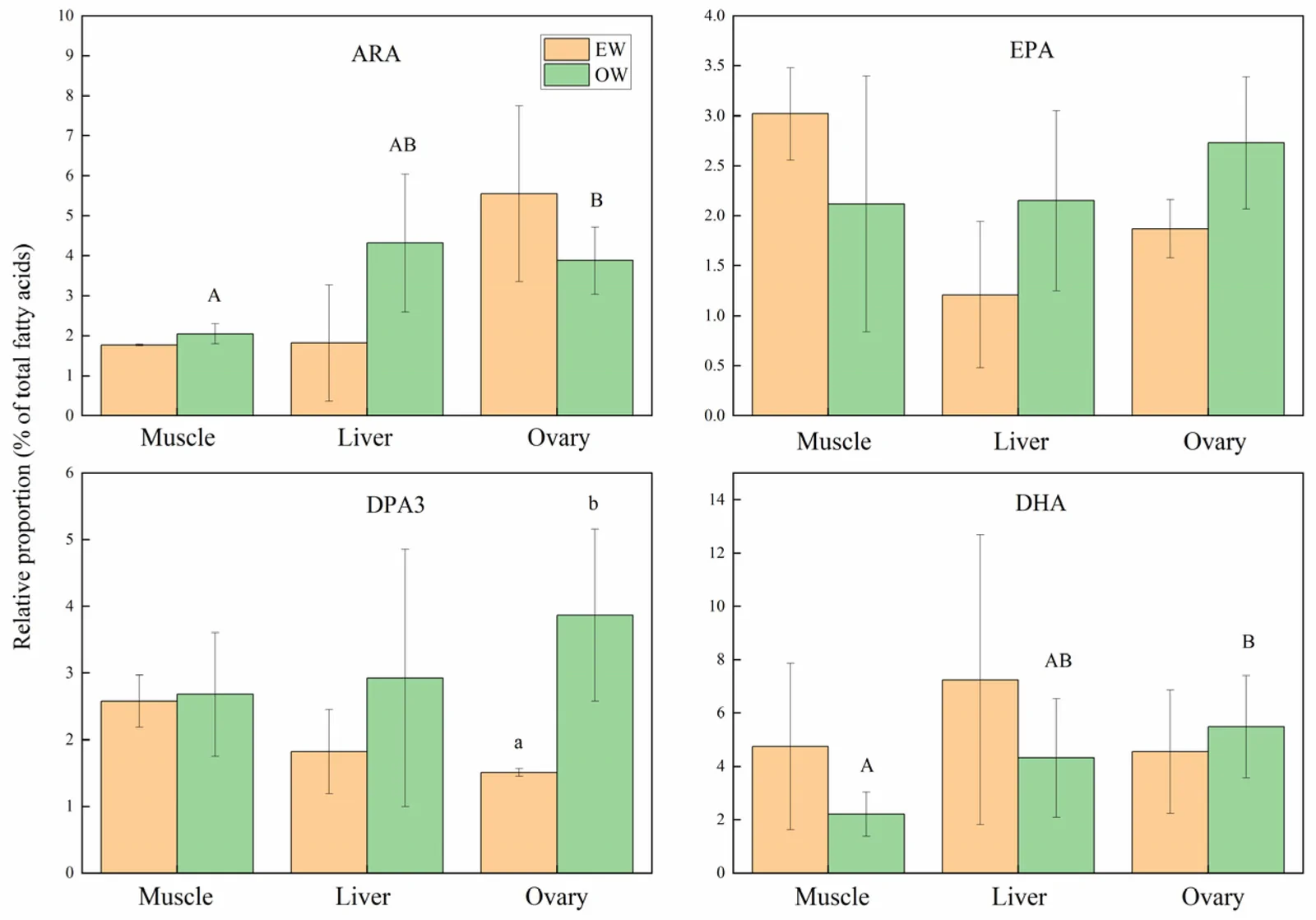
Comparison of the main fatty acid contents of PUFA (ARA, EPA, DPA3, and DHA) in different tissues of Japanese eels (*A. japonica*) from EW and OW. EW: estuary waters; OW: offshore waters. Error bars represent standard deviation (SD). Different small letters (a, b) denote significant differences between samples from different waters of EW and OW. Different capital letters (A, B) denote significant differences among different tissues of muscle, liver and ovary.

Figure 4 presents the tissue distribution of the main PUFA (ARA, EPA, DPA3, and DHA) in *A. japonica* from EW and OW. For ARA, EW fish showed the highest content in the ovary and lowest in the muscle, with no significant inter-tissue differences (*p* > 0.05); in contrast, OW fish had the highest in the liver and lowest in the muscle, with muscle content significantly lower than that in the ovary (*p* < 0.05). For EPA, EW fish exhibited the highest in the muscle and lowest in the liver, whereas OW fish showed the highest in the ovary and lowest in the muscle; no significant differences were found among tissues within either group (*p* > 0.05). For DPA3, EW fish had the highest in the muscle and lowest in the ovary; in OW fish, the highest was in ovary and lowest in muscle, and the ovarian DPA3 content in OW fish was significantly higher than that in EW fish (*p* < 0.05). For DHA, EW fish showed the highest in the liver and lowest in the ovary, while OW fish had the highest in the ovary and lowest in the muscle, with ovarian DHA significantly higher than muscle DHA (*p* < 0.05).

**Figure 5:**
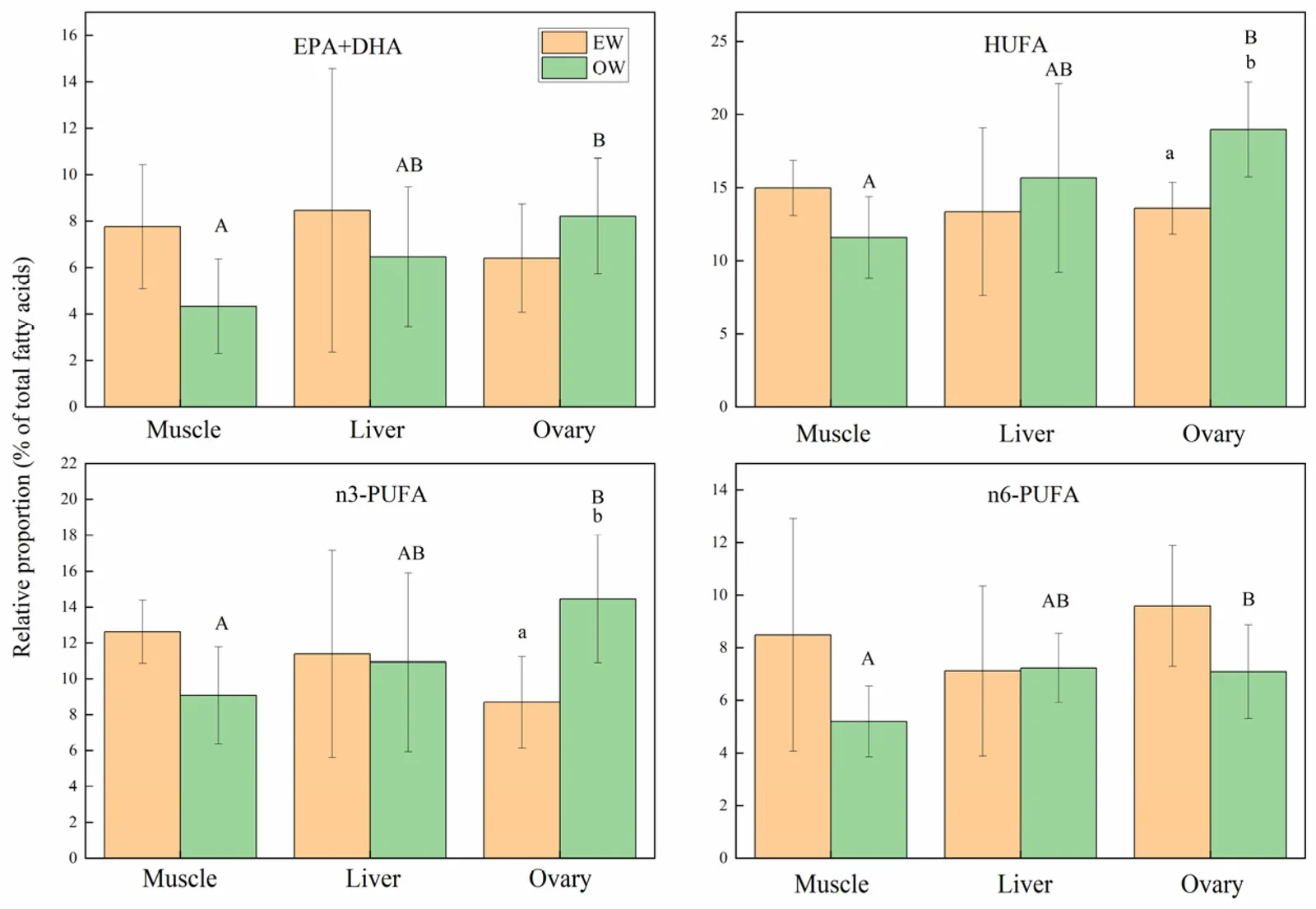
Comparison of the content of the important fatty acid combinations (DHA+EPA, HUFA, n3-PUFA, and n6-PUFA) in different tissues of Japanese eels (*A. japonica*) from EW and OW. EW: estuary waters; OW: offshore waters. Error bars represent standard deviation (SD). Different small letters (a, b) denote significant differences between samples from different waters of EW and OW. Different capital letters (A, B) denote significant differences among different tissues of muscle, liver and ovary.

Figure 5 presents the tissue distribution of important fatty acid combinations (DHA+EPA, HUFA, n3-PUFA, and n6-PUFA) in *A. japonica* from EW and OW. For DHA+EPA, EW fish showed the highest content in liver and lowest in ovary, with no significant inter-tissue differences (*p* > 0.05); in contrast, OW fish had the highest in the ovary and lowest in the muscle, with muscle content significantly lower than the ovary (*p* < 0.05). For HUFA, EW fish exhibited the highest in the muscle and lowest in the liver, with no significant inter-tissue differences (*p* > 0.05), whereas OW fish showed the highest in the ovary and lowest in the muscle, and the ovarian HUFA content in OW fish was significantly higher than that in EW fish (*p* < 0.05). For n3-PUFA, EW fish had the highest in the muscle and lowest in the ovary, with muscle content significantly lower than the ovary (*p* > 0.05), while OW fish had the highest in the ovary and lowest in the muscle, and the ovarian n3-PUFA content in OW fish was significantly higher than that in EW fish (*p* < 0.05). For n6-PUFA, EW fish showed the highest in the ovary and lowest in the liver, whereas OW fish had the highest in the liver and lowest in the muscle, with muscle content significantly lower than the ovary (*p* < 0.05).

**Figure 6:**
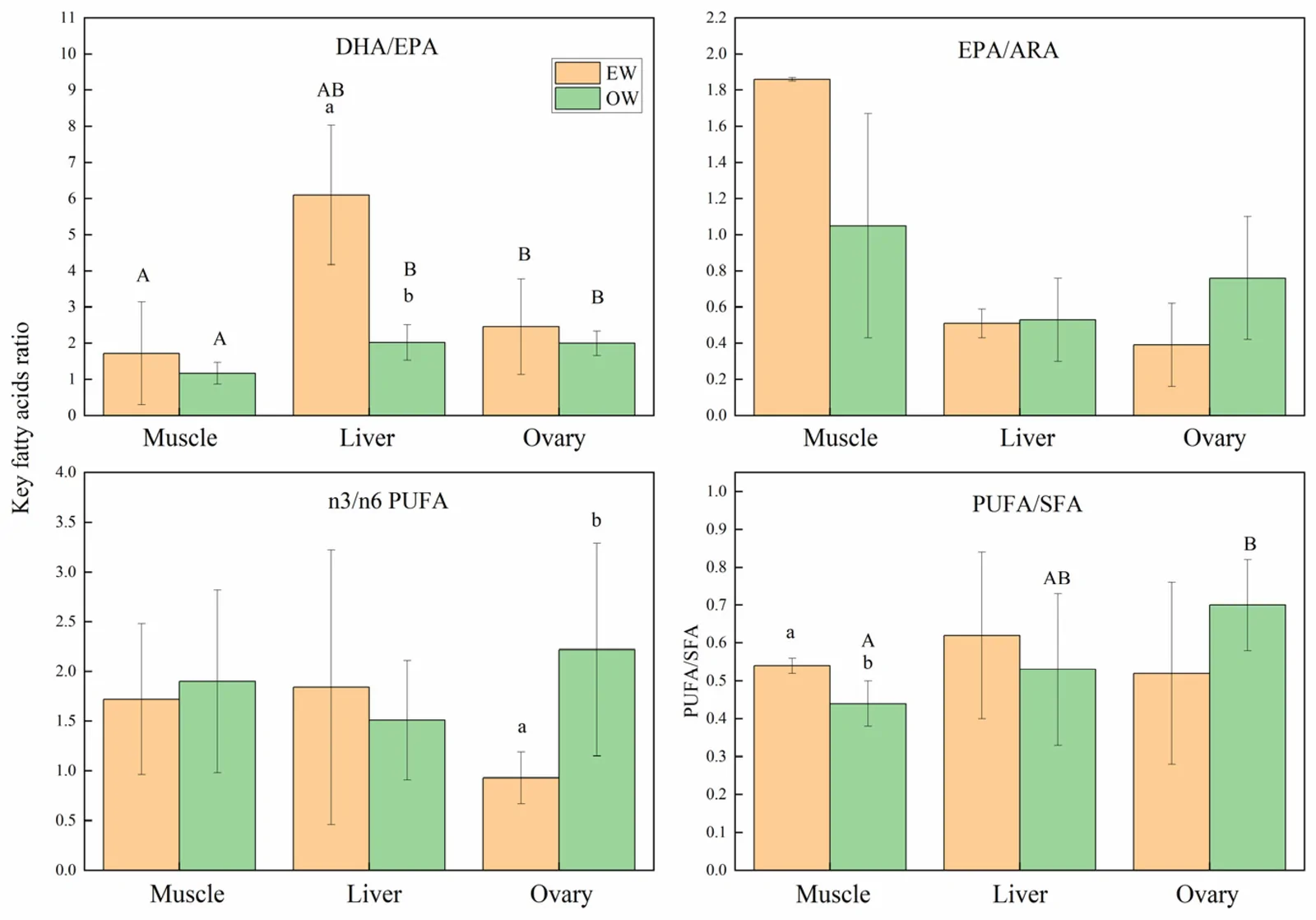
Comparison of the key fatty acid ratios (DHA/EPA, EPA/ARA, n3/n6 PUFA, and PUFA/SFA) in different tissues of Japanese eels (*A. japonica*) from EW and OW. EW: estuary waters; OW: offshore waters. Error bars represent standard deviation (SD). Different small letters (a, b) denote significant differences between samples from different waters of EW and OW. Different capital letters (A, B) denote significant differences among different tissues of muscle, liver and ovary.

Figure 6 presents the key fatty acid ratios (DHA/EPA, EPA/ARA, n3/n6 PUFA, and PUFA/SFA) in different tissues of *A. japonica* from EW and OW. For DHA/EPA, both groups showed the highest ratio in the liver and the lowest in the muscle. In EW fish, muscle DHA/EPA was significantly lower than that in the ovary (*p* < 0.05); in OW fish, muscle DHA/EPA was significantly lower than both the liver and ovary (*p* < 0.05). For EPA/ARA, both groups had the highest ratio in muscle. In EW fish, the lowest ratio was in the ovary, whereas in OW fish, the lowest ratio was in the liver; no significant differences were found among tissues within either group (*p* > 0.05). For n3/n6 PUFA, EW fish showed the highest in the liver and lowest in the ovary, with no significant inter-tissue differences (*p* > 0.05), whereas OW fish had the highest in the ovary and lowest in the liver, with the ovarian ratio significantly higher in OW than in EW (*p* < 0.05). For PUFA/SFA, EW fish had the highest in the liver and lowest in the ovary, with no significant inter-tissue differences (*p* > 0.05), while OW fish showed the highest in the ovary and lowest in the muscle, and the muscle ratio in EW fish was significantly higher than that in OW fish (*p* < 0.05).

## 4. Discussion

### 4.1. Fatty Acid Composition Characteristics in Different Tissues and the Manifestation of Tissue-Specific Functions

Muscle is the primary tissue for energy storage, and SFA and MUFA are considered the preferred fatty acids for oxidation to provide energy [27]. Studies have shown that, in both freshwater and marine fish, C16:0 and C18:1n9c are typically the most abundant fatty acids in muscle [28]. The fatty acid composition of muscle from both EW and OW eels in this study followed this pattern, consistent with the fundamental roles of these two fatty acids in energy provision and maintenance of muscle cell membrane function. In terms of tissue distribution, C16:0 was highest in the muscle of EW eels, whereas C18:1n9c was highest in the muscle of OW eels (Figure 3), suggesting a possible association between muscle fatty acid composition and habitat-related metabolic demands. Additionally, during early gonadal development, muscle also stores considerable amounts of n3-PUFA. In the muscle of *Illex argentinus*, DHA is the most abundant PUFA, followed by EPA [29], a pattern consistent with the distribution of DHA and EPA in the muscle of EW eels in this study. This is consistent with the interpretation that before seaward entry, Yangtze River Estuary eels store substantial amounts of DHA and EPA in their muscle. Research on European whitefish has shown that DHA and EPA contents in muscle decrease by approximately 60% from late summer to the spawning period [30]. In the present study, the DHA/EPA ratio in the muscle of offshore eels decreased by 23% compared with that of estuarine eels (Table 1), which may reflect utilization of muscle DHA during reproductive progression, potentially in support of gonadal development.

Liver is the central organ for lipid metabolism in fish, responsible for the synthesis, transformation, and distribution of fatty acids to other tissues [31]. The liver is not only rich in C18:1n9c but also contains abundant precursors of ARA for synthesis and metabolism. During fish reproductive migration, the ARA metabolic pathway in the liver is significantly activated, participating in environmental adaptation such as osmoregulation and gonadal development [32]. In this study, the ARA content in the liver of OW eels was higher than that in EW eels (Table 2), suggesting a difference in hepatic ARA levels between the two groups that may be associated with distinct habitat-related metabolic demands. Compared with muscle, the liver often contains a higher proportion of DHA, reflecting tissue-specific functional specialization [33,34,35]. In *Paralichthys olivaceus*, the DHA/EPA ratio in the liver of broodstock at gonadal stages III–IV was significantly higher than that at stage V, which has been interpreted as the liver playing a storage role for DHA during early gonadal development [33]. In the present study, the DHA/EPA ratio in the liver of EW eels was the highest among tissues and significantly higher than that in OW eels (Figure 6). This pattern is consistent with the expected hepatic DHA storage function during the early stage of gonadal development in eels prior to seaward transition. The n3/n6 PUFA ratio is considered a useful indicator of tissue functional orientation and metabolic homeostasis [36,37]. In *Tridentiger trigonocephalus* from the Yangtze Estuary, the n3/n6 PUFA ratio in the liver reached 8.05, higher than that in the ovary (7.00) and muscle (6.44) [38]. A similar pattern was observed in the liver of EW eels, where this ratio was highest among the three tissues. In contrast, in *Pampus argenteus* from offshore waters, the n3/n6 PUFA ratio in the gonad was significantly higher than that in the liver and muscle [39], a pattern comparable to that observed in the offshore eels in this study. Specifically, in EW eels at early gonadal development, the n3/n6 ratio was highest in the liver (Table S1, Figure 6); as gonadal development progressed, the ovarian n3/n6 ratio in OW eels reached the maximum among the three tissues (Table S2, Figure 6) and became significantly higher in OW eels than in EW eels (Table 3, Figure 6). The high n3/n6 ratio in the liver of estuarine eels is consistent with its role as a lipid metabolic hub, whereas the elevated ratio in the ovary of offshore eels may be associated with the nutritional demands of advancing gonadal development. Taken together, the hepatic fatty acid profiles observed in this study are consistent with the liver’s central role in lipid metabolism and temporary storage of DHA and n3-PUFA during early gonadal development. The shift in n3/n6 ratio from liver in EW eels to ovary in OW eels further suggests a dynamic re-distribution of n3-PUFA toward reproductive tissues as gonadal maturation progresses, although these associations remain correlational and require experimental validation.

During reproduction, fish preferentially transport and store critical HUFA in the developing gonad [40]. This is supported by findings in wild *Paralichthys olivaceus* broodstock, where stage V eggs contained large amounts of n3-HUFA, with proportions of EPA, DPA3, and DHA significantly higher than those in muscle and liver at the same developmental stage [33], suggesting that fish coordinate fatty acid mobilization across tissues to supply HUFA to the ovaries during gonadal maturation. The DHA/EPA ratio is often used as an indicator of tissue-specific DHA demand [41]. In *Tridentiger trigonocephalus*, the ovarian DHA/EPA ratio (1.59) was higher than that in muscle [38]; a pattern that is even more pronounced in bluefin tuna, where the ovarian DHA/EPA ratio reaches 4.5 [42]. In the present study, the ovarian DHA/EPA ratio in both groups of eels was higher than that in muscle, being 1.4- and 1.7-fold higher (Tables S1 and S2), respectively, which is consistent with a preferential distribution of DHA toward ovarian tissues. Similarly, the n3/n6 PUFA ratio in the gonad was relatively high and significantly higher than that in the liver and muscle [38,39], suggesting that n3-PUFA tend to be preferentially utilized by developing germ cells. In this study, the ovarian n3/n6 ratio in OW eels was significantly higher than that in EW eels (Table 3, Figure 6), with higher n3-HUFA proportions observed in the ovaries of OW eels (Table 3, Figure 5). Overall, the contents of DPA3, HUFA, and n3-PUFA, as well as the n3/n6 PUFA ratio, in the ovary of OW eels were significantly higher than those in EW eels (Table 3).

Taken together, the tissue-specific fatty acid profiles observed in this study are consistent with a functional division among the muscle, the liver, and ovary in *A. japonica*. Muscle appears to serve primarily as a reservoir for energy-providing SFA or MUFA and stored n3-PUFA that may be mobilized during reproduction; the liver functions as the central hub for fatty acid metabolism and temporary storage, particularly of DHA and n3 or n6 intermediates; and ovary acts as the final destination for selectively enriched HUFA, especially DHA and n3-PUFA, which are essential for oocyte quality and embryonic development. The progressive shift in n3- and n6-PUFA distribution from liver in estuarine eels to ovary in offshore eels is consistent with the view that fatty acid allocation is associated with reproductive stage, with the ovary showing increasing priority for HUFA deposition as gonadal maturation advances. However, these associations remain correlational, and the confounding effects of habitat, size, and developmental stage cannot be ruled out.

### 4.2. Fatty Acids Distribution Pattern in Different Tissues and Its Roles in Energy Supply and Gonadal Development

Different fatty acids play distinct and well-defined roles in the energy supply and reproductive processes of fish [7,43]. SFA and MUFA serve as the primary energy-providing fatty acids. When fish face periods of high energy demand, such as starvation, migration or reproduction, these fatty acids are preferentially mobilized to provide energy through β-oxidation [44]. Gonadal development requires substantial energy consumption, and major fatty acids such as C16:0 and C18:1n9c can provide large amounts of energy and carbon skeletons for the growth and maturation of oocytes [28]. Particularly during the early life stages, fish rely entirely on nutrients from the yolk for energy. At this stage, C16:0, C18:0 and C18:1n9c are preferentially used to provide ATP for embryo development and larval hatching [45]. In this study, compared with offshore eels, Yangtze Estuary eels had a higher C16:0 content in the ovary, while offshore eels had higher contents of C18:0, C16:1 and C18:1n9c in the ovary (Table 3, Figure 3). The PUFA/SFA ratio is often considered an indicator of energy allocation strategy in fish. A high PUFA/SFA ratio confers optimal fluidity and functionality to oocyte membranes, while the stored PUFA also provides essential structural components and energy sources for early embryonic development [42]. The low PUFA/SFA in muscle and the liver is consistent with their roles as energy storage and metabolic tissues, whereas the higher PUFA/SFA in the gonad may indicate the importance of PUFA for reproductive tissues [38,46]. In this study, the PUFA/SFA in the muscle and liver of offshore eels was relatively low, while it was highest in the ovary (Table S2, Figure 6). This pattern is consistent with an energy allocation strategy in which the muscle and liver tend to prioritize the storage of SFA and MUFA as readily oxidizable energy reserves, whereas the ovary shows progressive enrichment of PUFA, suggesting that PUFA/SFA may serve as a correlative indicator of reproductive-driven allocation. It should be noted that the above interpretation of energy allocation is derived exclusively from relative proportions of fatty acids, and thus represents a qualitative summary of compositional trends rather than a quantitative assessment based on absolute fatty acid concentrations.

ARA is a precursor for the synthesis of eicosanoids such as prostaglandins, directly participating in the regulation of reproductive processes including ovulation and spawning [7]. Studies on *Cynoglossus semilaevis* have shown that dietary ARA levels significantly affect estradiol (E_2_) synthesis in females, which is consistent with the enrichment of the ARA pathway observed in fish during reproductive migration [47]. Beyond ARA, the DHA/EPA ratio is also crucial for reproductive success [41]. Studies on artificial reproduction of *A. japonica* found that the gonadosomatic index (GSI) showed the strongest negative correlation with muscle DHA content, suggesting that DHA stored in the muscle may be mobilized toward the gonad during gonadal development [48]. In the present study, DHA distribution across tissues differed between EW and OW eels. In EW eels, DHA was highest in the liver and lowest in the muscle; in OW eels, DHA was highest in the ovary and lowest in the muscle (Table S1, Figure 4). Similar redistribution patterns of EPA and DHA between the muscle and ovary have been observed in artificially matured European eel [49], where the combined content of EPA and DHA decreased in muscle but increased in ovaries during hormonal stimulation, suggesting that preferential allocation of these n3-PUFA to reproductive tissues may be a conserved feature across *Anguilla* species. DHA is essential for the development of the fish brain and retina [50], and a high DHA/EPA ratio is critical for larval brain and retinal development, which in turn affects larval survival, growth rate, and predatory ability [51]. Accordingly, during gonadal development, fish are thought to regulate the DHA/EPA ratio to ensure sufficient DHA transfer to the ovary [52]. In *Paralichthys olivaceus* broodstock, the hepatic DHA/EPA ratio at gonadal stages III–IV was significantly higher than that at stage V, while the EPA/ARA ratio in eggs was significantly lower than that at stage V [33]. A similar pattern was observed in the present study: the hepatic DHA/EPA ratio was higher in EW eels (early gonadal development) than in OW eels (Table 2, Figure 6), whereas the ovarian EPA/ARA ratio was lower in EW eels than in OW eels (Table 3, Figure 6). These changes in ratios may reflect stage-specific differences in the relative abundance of DHA, EPA, and ARA in ovarian tissues. The extremely low EPA/ARA ratio in the gonad is thought to ensure sufficient ARA availability for eicosanoid synthesis, as excessive EPA can inhibit the conversion of ARA into active hormones [46]. Therefore, maintaining an appropriate EPA/ARA ratio is considered crucial for normal ovulation and fertilization success [7]. Collectively, the coordinated changes in DHA/EPA and EPA/ARA ratios between estuarine and offshore eels are consistent with a stage-dependent pattern: at early gonadal development (EW, stage II), the liver shows a high DHA/EPA ratio, consistent with a storage role; whereas during progressive gonadal development (OW, stage III), hepatic DHA/EPA decreases while ovarian DHA and EPA increase and ARA decreases. These associations may reflect a dual priority—ARA for eicosanoid-mediated reproductive hormone signaling and DHA for the nutritional demands of ovarian development—although causal inferences cannot be drawn from the present data.

The n3/n6 PUFA ratio fundamentally affects egg and larval quality, playing a crucial role in maintaining normal reproductive cycles, stable sex hormone synthesis, and the healthy development of gametes [53]. In this study, the n3/n6 ratio was highest in the liver of estuarine eels (Table S1, Figure 6), whereas it was highest in the ovary of offshore eels (Table S2, Figure 6), with the ovarian ratio significantly higher in OW than in EW (Table 3, Figure 6), consistent with a redistribution of the predominant tissue for this ratio between the two groups. The observed tissue-level shift in the n3/n6 ratio from the liver to the ovary is consistent with, but does not prove, an active metabolic re-orientation that prioritizes n3-PUFA deposition in reproductive tissues in association with the progression from estuarine to offshore habitats. Taken together, the ratio-based evidence supports the view that fatty acid metabolism in *A. japonica* shows patterns associated with reproductive stage: SFA and MUFA tend to dominate somatic energy stores, while HUFA—particularly DHA and n3-PUFA—are found at higher concentrations in the ovary, which may be linked to gonadal development and maturation. Nevertheless, the correlational nature of these findings must be emphasized, and the confounding of habitat, size, and gonadal stage precludes any direct attribution of the observed patterns to a single factor.

## 5. Conclusions

This study provides a descriptive characterization of fatty acid profiles in the muscle, liver, and ovary of *A. japonica* collected from the Yangtze Estuary (EW) and offshore waters (OW), revealing tissue-specific distribution patterns and population-level differences that are associated with differences in habitat, reproductive stage, and body size. The key patterns observed include: (1) the muscle shows higher SFA proportions in estuarine eels and higher MUFA proportions in offshore eels, a difference consistent with the tissue’s role as an energy storage depot; (2) the liver exhibits high DHA/EPA and n3/n6 ratios in estuarine eels, which may be associated with early gonadal development; and (3) the ovary shows progressive accumulation of HUFA, particularly DHA and n3-PUFA, in offshore eels, with higher ovarian n3-PUFA proportions and n3/n6 ratios compared with estuarine eels. The most notable population-level distinction lies in the ovarian fatty acid composition: EW ovaries are characterized by higher proportions of C16:0 and ARA, whereas OW ovaries show enrichment in n3-PUFA (EPA, DPA3, and DHA) and elevated n3/n6 ratios. These patterns are consistent with the hypothesis that fatty acid allocation is associated with the estuarine-to-offshore transition and reproductive progression; however, because the EW and OW groups differ simultaneously in multiple factors (location, gonadal stage, and size), these associations cannot be interpreted as causal. From an ecological and applied perspective, these findings provide baseline data that may inform future studies on the reproductive physiology of *A. japonica* and the conservation of its spawning populations.

## Figures and Tables

**Table 1:** Fatty acid composition in the muscles of Japanese eels (*A. japonica*) from EW and OW (mean ± SD, *n* = 6, %).

Fatty Acid	EW	OW	Test	T or U	*p*
C10:0	0.01 ± 0.00	0.01 ± 0.00	*t*	1.598	0.145
C12:0	0.16 ± 0.04 ^a^	0.08 ± 0.03 ^b^	MW	2.000	0.010
C13:0	0.08 ± 0.04	0.05 ± 0.02	MW	8.000	0.109
C14:0	4.00 ± 1.17	3.60 ± 0.38	*t*	0.808	0.438
C15:0	0.88 ± 0.33	0.99 ± 0.68	*t*	−0.341	0.741
C16:0	24.58 ± 7.01	22.42 ± 0.51	Welch	0.750	0.487
C17:0	0.78 ± 0.21	0.81 ± 0.08	*t*	−0.295	0.774
C18:0	5.67 ± 1.73	5.38 ± 0.56	*t*	0.392	0.704
C20:0	0.25 ± 0.05	0.22 ± 0.03	*t*	1.089	0.302
C21:0	0.08 ± 0.01	0.11 ± 0.06	Welch	−1.486	0.194
C22:0	0.11 ± 0.03	0.10 ± 0.07	Welch	0.278	0.789
C23:0	0.14 ± 0.05	0.19 ± 0.12	*t*	−0.711	0.504
SFA	36.75 ± 9.36	33.96 ± 1.62	*t*	0.719	0.488
C14:1	0.23 ± 0.07	0.19 ± 0.06	*t*	0.938	0.370
C15:1	0.05 ± 0.01 ^a^	0.01 ± 0.00 ^b^	*t*	8.245	<0.001
C16:1	9.81 ± 1.59	10.13 ± 1.85	*t*	−0.317	0.757
C17:1	0.91 ± 0.18	0.83 ± 0.43	*t*	0.413	0.689
C18:1n9t	0.32 ± 0.07 ^a^	0.44 ± 0.11 ^b^	*t*	−2.483	0.032
C18:1n9c	35.74 ± 2.74	37.80 ± 5.58	*t*	−0.752	0.471
C20:1n9	1.79 ± 0.56	1.58 ± 0.36	*t*	0.753	0.469
C22:1n9	0.13 ± 0.03 ^a^	0.08 ± 0.02 ^b^	*t*	2.772	0.020
C24:1n9	0.05 ± 0.03	0.05 ± 0.02	*t*	−0.412	0.689
MUFA	42.70 ± 13.52	51.11 ± 3.79	*t*	−1.467	0.173
C18:2n6c	4.99 ± 3.08	2.67 ± 1.35	*t*	1.687	0.122
C20:2	0.91 ± 0.26 ^a^	0.60 ± 0.14 ^b^	*t*	2.664	0.024
C22:2	0.06 ± 0.02	0.05 ± 0.04	*t*	0.290	0.778
C18:3n6	0.14 ± 0.12	0.10 ± 0.06	*t*	0.767	0.461
C18:3n3(ALA)	2.11 ± 0.41	1.70 ± 0.60	*t*	1.388	0.195
C20:3n6	0.45 ± 0.22	0.38 ± 0.17		0.627	0.545
C20:3n3	0.51 ± 0.05	0.36 ± 0.18	*t*	1.880	0.093
C20:4n6(ARA)	1.83 ± 0.33	2.05 ± 0.25	*t*	−1.299	0.223
C20:5n3(EPA)	2.88 ± 0.71	2.12 ± 1.28	*t*	1.255	0.238
C22:5n3(DPA3)	2.56 ± 0.47	2.68 ± 0.93	*t*	−0.293	0.776
C22:6n3 (DHA)	4.01 ± 2.24	2.21 ± 1.28	*t*	1.842	0.095
PUFA	20.36 ± 4.26 ^a^	14.93 ± 2.43 ^b^	MW	0.000	0.004
EPA + DHA	6.89 ± 2.18	4.34 ± 2.04	*t*	2.093	0.063
HUFA	14.39 ± 1.75	11.60 ± 2.79	*t*	2.084	0.064
n3-PUFA	11.98 ± 1.81	9.08 ± 2.72	*t*	2.181	0.054
n6-PUFA	7.42 ± 3.20	5.20 ± 1.35	*t*	1.565	0.149
DHA/EPA	1.52 ± 1.00	1.17 ± 0.30	*t*	0.821	0.431
EPA/ARA	1.62 ± 0.46	1.05 ± 0.62	*t*	1.841	0.095
n3/n6 PUFA	1.83 ± 0.74	1.90 ± 0.92	*t*	−0.158	0.877
PUFA/SFA	0.56 ± 0.03 ^a^	0.44 ± 0.06 ^b^	Welch	4.624	0.003

Values in the same row with different superscript letters (a, b) mean significant difference (*p* < 0.05). T or U: value of the test statistic; data in column T correspond to the t-values from Student’s *t*-test (*t*) and Welch’s *t*-test (Welch), and data in column U correspond to the U-values from the Mann–Whitney (MW) test. *p* is the corresponding probability value. The same applies to Table 2 and Table 3.

**Table 2:** Fatty acid composition in the liver of Japanese eels (*A. japonica*) from EW and OW (mean ± SD, *n* = 6, %).

Fatty Acid	EW	OW	Test	T or U	*p*
C6:0	0.02 ± 0.03	0.01 ± 0.01	MW	4.500	0.558
C11:0	0.03 ± 0.01	0.05 ± 0.06	MW	4.000	0.825
C12:0	0.07 ± 0.02	0.06 ± 0.04	*t*	0.777	0.457
C13:0	0.05 ± 0.03	0.03 ± 0.02	*t*	1.197	0.276
C14:0	2.72 ± 0.84	2.39 ± 0.74	*t*	0.718	0.489
C15:0	0.40 ± 0.22	0.75 ± 0.46	Welch	−1.693	0.134
C16:0	21.35 ± 2.67 ^a^	25.21 ± 2.88 ^b^	*t*	−2.408	0.037
C17:0	0.48 ± 0.22	0.73 ± 0.17	*t*	−2.229	0.050
C18:0	7.13 ± 1.88	7.09 ± 1.65	*t*	0.039	0.969
C20:0	0.22 ± 0.09	0.22 ± 0.05	*t*	0.113	0.913
C21:0	0.05 ± 0.02	0.09 ± 0.06	Welch	−1.315	0.206
C22:0	0.05 ± 0.02	0.10 ± 0.07	Welch	−1.401	0.302
SFA	32.59 ± 2.86 ^a^	37.04 ± 3.94 ^b^	*t*	−2.237	0.049
C14:1	0.12 ± 0.04	0.10 ±0.05	*t*	0.856	0.412
C16:1	6.02 ± 1.87	7.56 ± 1.71	*t*	−1.490	0.167
C17:1	0.48 ± 0.19	0.75 ± 0.43	*t*	−1.302	0.225
C18:1n9t	0.30 ± 0.05 ^a^	0.49 ± 0.09 ^b^	Welch	−4.404	0.002
C18:1n9c	36.81 ± 7.36	33.79 ± 3.05	*t*	0.927	0.376
C20:1n9	1.80 ± 0.41 ^a^	1.23 ± 0.26 ^b^	*t*	2.882	0.016
C22:1n9	0.15 ± 0.05 ^a^	0.09 ± 0.03 ^b^	*t*	2.543	0.032
MUFA	45.70 ± 8.43	44.03 ± 4.56	*t*	0.427	0.679
C18:2n6c	4.27 ± 1.86	2.48 ± 1.06	*t*	2.045	0.068
C20:2	0.71 ± 0.20	0.75 ± 0.20	MW	17.000	0.873
C22:2	0.07 ± 0.04	0.05 ± 0.02	*t*	1.022	0.354
C18:3n6	0.09 ± 0.05	0.06 ± 0.03	*t*	1.387	0.196
C18:3n3(ALA)	0.90 ± 0.51	1.17 ± 0.26	*t*	−1.165	0.271
C20:3n6	0.53 ± 0.26	0.37 ± 0.09	*t*	1.360	0.204
C20:3n3	0.24 ± 0.17	0.35 ± 0.10	*t*	−1.259	0.240
C20:4n6(ARA)	2.72 ± 2.05	4.32 ± 1.72	*t*	−1.410	0.192
C20:5n3(EPA)	1.76 ± 0.93	2.15 ± 0.90	*t*	−0.751	0.470
C22:5n3(DPA3)	1.79 ± 0.46	2.93 ± 1.93	Welch	−1.400	0.215
C22:6n3 (DHA)	8.88 ± 7.20	4.32 ± 2.23	Welch	1.484	0.189
PUFA	21.44 ± 8.51	18.93 ± 5.61	*t*	0.602	0.561
EPA+DHA	10.64 ± 8.01	6.47 ± 3.02	*t*	1.194	0.260
HUFA	16.42 ± 9.51	15.67 ± 6.46	*t*	0.159	0.877
n3-PUFA	13.53 ± 7.89	10.92 ± 4.98	*t*	0.687	0.508
n6-PUFA	7.16 ± 2.12	7.23 ± 1.32	*t*	−0.072	0.944
DHA/EPA	5.03 ± 2.29 ^a^	2.02 ± 0.49 ^b^	Welch	3.154	0.022
EPA/ARA	0.70 ± 0.26	0.53 ± 0.23	*t*	1.118	0.292
n3/n6 PUFA	1.97 ± 1.18	1.51 ± 0.60	MW	14.000	0.522
PUFA/SFA	0.66 ± 0.27	0.53 ± 0.20	*t*	0.990	0.345

Values in the same row with different superscript letters (a, b) mean significant difference (*p* < 0.05).

**Table 3:** Fatty acid composition in the ovary of Japanese eels (*A. japonica*) from EW and OW (mean ± SD, *n* = 6, %).

Fatty Acid	EW	OW	Test	T or U	*p*
C12:0	0.21 ± 0.24	0.02 ± 0.01	Welch	1.124	0.462
C13:0	0.56 ± 0.50	0.03 ± 0.03	Welch	1.832	0.208
C14:0	2.83 ± 0.35 ^a^	1.24 ± 0.45 ^b^	*t*	5.276	0.001
C15:0	1.11 ± 0.87	0.49 ± 0.30	*t*	1.646	0.144
C16:0	24.38 ± 3.93 ^a^	11.25 ± 2.91 ^b^	*t*	5.743	0.001
C17:0	0.99 ± 0.75	0.41 ± 0.09	Welch	1.322	0.315
C18:0	8.53 ± 2.93 ^a^	15.40 ± 3.93 ^b^	*t*	−2.648	0.033
C20:0	0.31 ± 0.21 ^a^	3.33 ± 0.14 ^b^	*t*	−22.224	<0.001
SFA	39.63 ± 9.96	31.62 ± 1.09	Welch	1.390	0.074
C14:1	0.52 ± 0.45	0.06 ± 0.01	Welch	1.753	0.222
C15:1	0.32 ± 0.43	0.61 ± 0.22	*t*	−1.307	0.239
C16:1	6.08 ± 1.34	8.52 ± 1.65	*t*	−2.198	0.064
C17:1	0.96 ± 0.04	1.30 ± 0.34	*t*	−1.317	0.245
C18:1n9t	0.40 ± 0.23	0.49 ± 0.10	*t*	−0.810	0.445
C18:1n9c	31.58 ± 8.74	34.34 ± 4.18	*t*	−0.665	0.527
C20:1n9	1.69 ± 0.26 ^a^	0.99 ± 0.31 ^b^	*t*	3.351	0.012
MUFA	40.75 ± 8.42	46.23 ± 2.28	Welch	−1.107	0.377
C18:2n6c	4.69 ± 2.76	2.50 ± 1.01	Welch	1.327	0.302
C20:2	0.84 ± 0.08 ^a^	0.57 ± 0.15 ^b^	*t*	2.790	0.027
C18:3n6	0.19 ± 0.04	0.16 ± 0.13	MW	2.000	0.826
C18:3n3 (ALA)	1.29 ± 0.29	2.01 ± 0.63	*t*	−1.853	0.113
C20:3n6	0.53 ± 0.16	0.50 ± 0.17	*t*	0.215	0.837
C20:4n6(ARA)	4.42 ± 2.50	3.88 ± 0.84	*t*	0.505	0.629
C20:5n3(EPA)	1.87 ± 0.29	2.73 ± 0.66	*t*	−2.098	0.074
C22:5n3(DPA3)	1.50 ± 0.09 ^a^	3.87 ± 1.29 ^b^	*t*	−2.457	0.049
C22:6n3 (DHA)	4.54 ± 2.31	5.49 ± 1.92	*t*	−0.652	0.535
PUFA	19.13 ± 3.98	22.16 ± 3.10	*t*	−1.269	0.245
EPA + DHA	6.41 ± 2.33	8.22 ± 2.48	*t*	−1.047	0.330
HUFA	13.60 ± 1.77 ^a^	18.99 ± 3.24 ^b^	*t*	−2.629	0.034
n3-PUFA	8.70 ± 2.55 ^a^	14.45 ± 3.55 ^b^	*t*	−2.465	0.043
n6-PUFA	9.59 ± 2.30	7.09 ± 1.78	*t*	1.816	0.112
DHA/EPA	2.46 ± 1.32	2.00 ± 0.34	Welch	0.598	0.607
EPA/ARA	0.54 ± 0.30	0.76 ± 0.34	*t*	−0.951	0.373
n3/n6 PUFA	0.93 ± 0.26 ^a^	2.22 ± 1.07 ^b^	Welch	−2.799	0.031
PUFA/SFA	0.52 ± 0.24	0.70 ± 0.12	*t*	−1.607	0.152

Values in the same row with different superscript letters (a, b) mean significant difference (*p* < 0.05).

## Data Availability

The data presented in this study are available in the article. Further information is available upon request from the corresponding author.

## Supplementary Materials

The following supporting information can be downloaded at https://www.mdpi.com/article/10.3390/ani16162568/s1: Figure S1. Comparison of ovarian histological sections between estuarine (EW) eels (A: Stage II, single-layered follicle (SLF) phase) and offshore eels (OW) (B: Stage III, adipose vesicle (AV) phase). Figure S2. Quantitative line profiles of Sr/Ca × 10^3^ ratios along otoliths of estuarine (EW) and offshore (OW) eels. Table S1: Key fatty acid composition and variation analysis among the muscle, liver and ovary of estuarine (EW) eels (estimated marginal mean ± SD, *n* = 6, %). Table S2: Key fatty acid composition and variation analysis among the muscle, liver and ovary of offshore (OW) eels (estimated marginal mean ± SD, *n* = 6, %).
