# Supplementary material for: Fatty Acid Profiles in Tissues of Japanese Eel (Anguilla japonica) from the Yangtze Estuary and Adjacent Offshore Waters: A Comparative Analysis
Source: Animals (Basel). 2026 Aug 18;16(16):2568. doi: 10.3390/ani16162568 (PMC13508901; doi:10.3390/ani16162568)
Supplement: Supplementary file 1 [file animals-16-02568-s001.zip › animals-4440289-supplementary.pdf]

Supplementary Materials

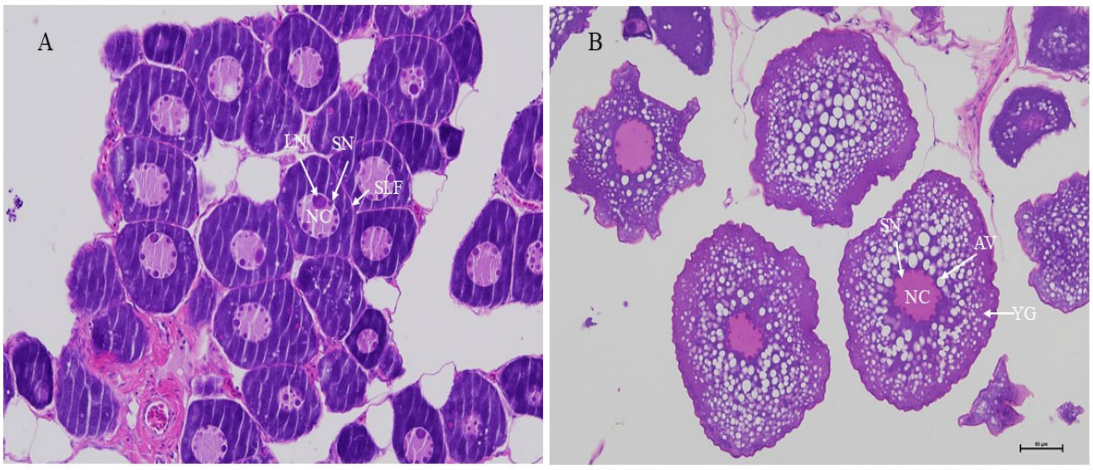

Figure S1. Comparison of ovarian histological sections between estuarine (EW) eels (A: Stage II, single-layered follicle (SLF) phase) and offshore (OW) eels (B: Stage III, adipose vesicle (AV) phase). NC: Nucleus; LN: Large nucleolus; SN: Small nucleolus; SLF: Single-layered follicle; AV: Adipose vesicle; YG: Yolk granule.

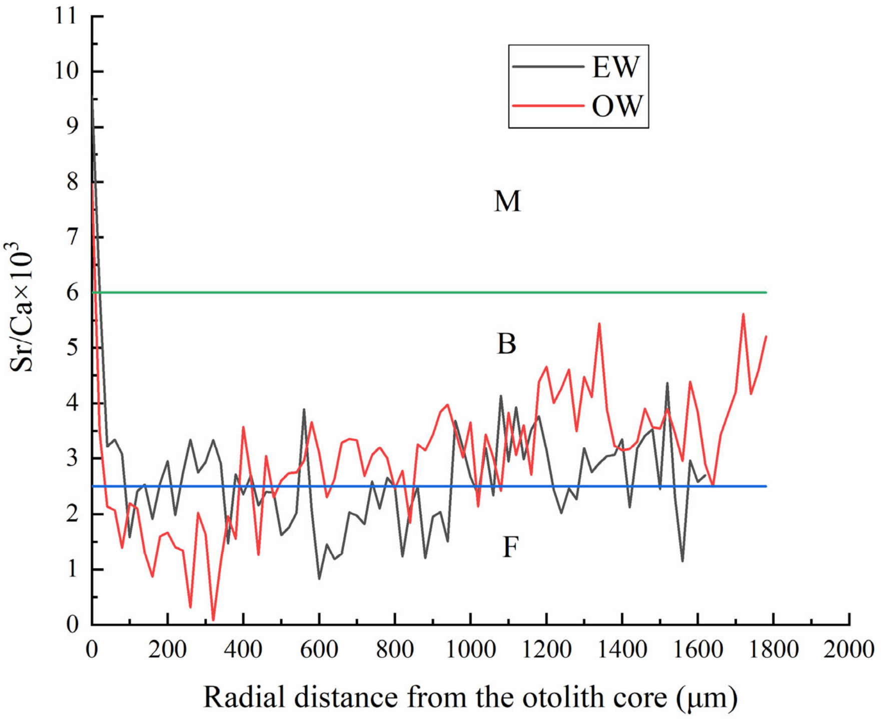

Figure S2. Quantitative line profiles of Sr/Ca×10^3 ratios along otoliths of estuarine (EW) and offshore (OW) eels. At the time of sampling, the estuarine group (EW) was at the stage of having dispersed from freshwater (F) into the brackish waters (B) of the estuary, whereas the offshore group was at the stage of having moved from freshwater (F), through the brackish waters (B) of the estuary, into the marine waters (M) of the offshore area. F: freshwater; B: brackish; M: marine.

Table S1. Key fatty acid composition and variation analysis among the muscle, liver and ovary of estuarine (EW) eels (Estimated marginal mean ± SD, *n*=6, %).

Fatty acid	Muscle	Liver	Ovary	Test of sphericity of Mauchly		In-subject effect test	
				Mauchly's W	P	F	P
SFA	41.36±12.43	31.40±3.03	39.63±9.96	0.100	0.317	0.686	0.554
MUFA	36.20±18.14	48.83±6.76	40.75±8.42	0.002	0.047	0.802	0.465
PUFA	22.28±5.86	19.37±6.47	19.13±3.98	0.252	0.502	0.963	0.456
C16:0	27.72±9.62	20.63±2.69	24.38±3.92	0.233	0.483	0.802	0.509
C18:0	6.69±2.06	7.03±1.85	8.53±2.93	0.001	0.023	0.382	0.600
C16:1	10.21±1.12	5.88±1.49	6.08±1.34	0.377	0.614	9.108	0.032
C18:1n9c	33.91±0.51	42.39±5.40	27.41±6.96	0.034	0.184	1.345	0.358
C20:4n6(ARA)	1.77±0.02	1.82±1.45	5.55±2.20	0.017	0.130	0.752	0.528
C20:5n3(EPA)	3.02±0.46	1.21±0.73	1.87±0.29	0.373	0.610	6.129	0.061
C22:5n3(DPA3)	2.58±0.39	1.82±0.63	1.51±0.06	0.383	0.619	3.778	0.120
C22:6n3 (DHA)	4.75±3.12	7.25±5.44	4.55±2.31	0.036	0.189	2.122	0.235
EPA+DHA	7.77±2.67	8.47±6.10	6.41±2.33	0.000	0.010	0.592	0.522
HUFA	14.98±1.89	13.36±5.73	13.60±1.77	0.417	0.646	0.266	0.779
n3-PUFA	12.63±1.76	11.40±5.77	8.70±2.55	0.014	0.119	1.749	0.285
n6-PUFA	8.49±4.42	7.12±3.23	9.59±2.30	0.001	0.032	0.465	0.566
DHA/EPA	$1.72±1.42^{A}$	$6.10±1.93^{AB}$	$2.46±1.32^{B}$	0.014	0.118	16.691	0.011
EPA/ARA	1.86±0.01	0.51±0.08	0.39±0.23	0.005	0.072	0.811	0.506
n3/n6	1.72±0.76	1.84±1.38	0.93±0.26	0.015	0.124	0.712	0.544
PUFA/SFA	0.54±0.02	0.62±0.22	0.52±0.24	0.883	0.940	0.257	0.786

Values are expressed as EM mean ± SD; Values in the same row different uppercase superscript letters mean significant difference (p<0.05). The same applies to Tables S2.

Table S2. Key fatty acid composition and variation analysis among the muscle, liver and ovary of offshore (OW) eels (Estimated marginal mean ± SD, *n*=6, %).

Fatty acid	Muscle	Liver	Ovary	Test of sphericity of Mauchly		In-subject effect test	
				Mauchly's W	P	F	P
SFA	33.96±1.62	37.04±3.94	31.62±1.09	0.632	0.400	6.569	0.015
MUFA	$51.11±3.79^{A}$	$44.03±4.56^{AB}$	$46.23±2.28^{B}$	0.403	0.162	6.050	0.019
PUFA	$14.93±2.43^{A}$	$18.93±5.61^{AB}$	$22.16±3.10^{B}$	0.110	0.012	4.647	0.080
C16:0	$22.42±0.51^{A}$	$25.21±2.88^{A}$	$11.25±2.91^{B}$	0.866	0.749	61.493	0.000
C18:0	$5.38±0.56^{A}$	$7.09±1.65^{A}$	$15.40±3.93^{B}$	0.367	0.135	11.536	0.003
C16:1	$10.13±1.85^{A}$	$7.56±1.71^{B}$	$8.52±1.65^{B}$	0.828	0.682	42.213	<0.001
C18:1n9c	37.80±5.58	33.79±3.05	34.34±4.18	0.415	0.172	2.124	0.170
C20:4n6(ARA)	$2.05±0.25^{A}$	$4.32±1.72^{AB}$	$3.88±0.84^{B}$	0.394	0.155	6.535	0.015
C20:5n3(EPA)	2.12±1.28	2.15±0.90	2.73±0.66	0.828	0.686	2.165	0.166
C22:5n3(DPA3)	2.68±0.93	2.93±1.93	3.87±1.29	0.490	0.240	1.368	0.298
C22:6n3 (DHA)	$2.21±0.83^{A}$	$4.32±2.23^{AB}$	$5.49±1.92^{B}$	0.268	0.072	6.117	0.018
EPA+DHA	$4.34±2.03^{A}$	$6.47±3.02^{AB}$	$8.22±2.48^{B}$	0.109	0.012	6.057	0.054
HUFA	$11.60±2.79^{A}$	$15.67±6.46^{AB}$	$18.99±3.24^{B}$	0.108	0.012	1.458	0.085
n3-PUFA	$9.08±2.71^{A}$	$10.92±4.98^{AB}$	$14.45±3.55^{B}$	0.124	0.015	4.017	0.097
n6-PUFA	$5.20±1.35^{A}$	$7.23±1.32^{AB}$	$7.09±1.78^{B}$	0.252	0.064	6.132	0.018
DHA/EPA	$1.17±0.30^{A}$	$2.02±0.49^{B}$	$2.00±0.34^{B}$	0.435	0.190	7.530	0.010
EPA/ARA	1.05±0.62	0.53±0.23	0.76±0.34	0.188	0.036	6.869	0.013
n3/n6	1.90±0.92	1.51±0.60	2.22±1.07	0.106	0.011	3.008	0.140
PUFA/SFA	$0.44±0.06^{A}$	$0.53±0.20^{AB}$	$0.70±0.12^{B}$	0.182	0.033	5.346	0.069
